# Supplementary material for: HIVprotI: an integrated web based platform for prediction and design of HIV proteins inhibitors
Source: J Cheminform. 2018 Mar 9;10:12. doi: 10.1186/s13321-018-0266-y (PMC5845081; doi:10.1186/s13321-018-0266-y)
Supplement: Supplementary file 2 — Additional file 2. Source code of HIVProtI web server. [file 13321_2018_266_MOESM2_ESM.zip › HIVprotI_Source-code/hivprotI/weka-3-6-6/doc/weka/filters/unsupervised/attribute/AddNoise.html]

AddNoise


---


|  |  |  |
| --- | --- | --- |
| **Overview** \| **Package** \| **Class** \| **Tree** \| **Deprecated** \| **Index** \| **Help** \| **Weka's home** | |  |
| **PREV CLASS**   **NEXT CLASS** | **FRAMES**    **NO FRAMES**     **All Classes** |  |
| SUMMARY: NESTED \| FIELD \| CONSTR \| METHOD | DETAIL: FIELD \| CONSTR \| METHOD |  |


---


## weka.filters.unsupervised.attribute Class AddNoise

```
java.lang.Object
  weka.filters.Filter
      weka.filters.unsupervised.attribute.AddNoise
```

**All Implemented Interfaces:**: java.io.Serializable, CapabilitiesHandler, OptionHandler, RevisionHandler, UnsupervisedFilter

---

``` public class AddNoise extends Filter implements UnsupervisedFilter, OptionHandler ```

An instance filter that changes a percentage of a given attributes values. The attribute must be nominal. Missing value can be treated as value itself.


Valid options are:

```
 -C <col>
  Index of the attribute to be changed 
  (default last attribute)
```

```
 -M
  Treat missing values as an extra value
```

```
 -P <num>
  Specify the percentage of noise introduced 
  to the data (default 10)
```

```
 -S <num>
  Specify the random number seed (default 1)
```

**Version:**
:   $Revision: 5543 $

**Author:**
:   Gabi Schmidberger (gabi@cs.waikato.ac.nz)

**See Also:**: Serialized Form

---

**Constructor Summary**

`AddNoise()`


| **Method Summary** | |
| --- | --- |
| `void` | `addNoise(Instances instances, int seed, int percent, int attIndex, boolean useMissing)`             add noise to the dataset a given percentage of the instances are changed in the way, that a set of instances are randomly selected using seed. |
| `java.lang.String` | `attributeIndexTipText()`             Returns the tip text for this property |
| `boolean` | `batchFinished()`             Signify that this batch of input to the filter is finished. |
| `java.lang.String` | `getAttributeIndex()`             Get the index of the attribute used. |
| `Capabilities` | `getCapabilities()`             Returns the Capabilities of this filter. |
| `java.lang.String[]` | `getOptions()`             Gets the current settings of the filter. |
| `int` | `getPercent()`             Gets the size of noise data as a percentage of the original set. |
| `int` | `getRandomSeed()`             Gets the random number seed. |
| `java.lang.String` | `getRevision()`             Returns the revision string. |
| `boolean` | `getUseMissing()`             Gets the flag if missing values are treated as extra values. |
| `java.lang.String` | `globalInfo()`             Returns a string describing this filter |
| `boolean` | `input(Instance instance)`             Input an instance for filtering. |
| `java.util.Enumeration` | `listOptions()`             Returns an enumeration describing the available options |
| `static void` | `main(java.lang.String[] argv)`             Main method for testing this class. |
| `java.lang.String` | `percentTipText()`             Returns the tip text for this property |
| `java.lang.String` | `randomSeedTipText()`             Returns the tip text for this property |
| `void` | `setAttributeIndex(java.lang.String attIndex)`             Sets index of the attribute used. |
| `boolean` | `setInputFormat(Instances instanceInfo)`             Sets the format of the input instances. |
| `void` | `setOptions(java.lang.String[] options)`             Parses a given list of options. |
| `void` | `setPercent(int newPercent)`             Sets the size of noise data, as a percentage of the original set. |
| `void` | `setRandomSeed(int newSeed)`             Sets the random number seed. |
| `void` | `setUseMissing(boolean newUseMissing)`             Sets the flag if missing values are treated as extra values. |
| `java.lang.String` | `useMissingTipText()`             Returns the tip text for this property |

**Methods inherited from class weka.filters.Filter**

`batchFilterFile, filterFile, getCapabilities, getOutputFormat, isFirstBatchDone, isNewBatch, isOutputFormatDefined, makeCopies, makeCopy, numPendingOutput, output, outputPeek, toString, useFilter, wekaStaticWrapper`

**Methods inherited from class java.lang.Object**

`equals, getClass, hashCode, notify, notifyAll, wait, wait, wait`

**Constructor Detail**

### AddNoise

```
public AddNoise()
```


**Method Detail**

### globalInfo

```
public java.lang.String globalInfo()
```

:   Returns a string describing this filter

    :   **Returns:**: a description of the filter suitable for displaying in the explorer/experimenter gui

---


### listOptions

```
public java.util.Enumeration listOptions()
```

:   Returns an enumeration describing the available options

    :   **Specified by:**: `listOptions` in interface `OptionHandler`
    :   **Returns:**: an enumeration of all the available options

---


### setOptions

```
public void setOptions(java.lang.String[] options)
                throws java.lang.Exception
```

:   Parses a given list of options.


    Valid options are:

    ```
     -C <col>
      Index of the attribute to be changed 
      (default last attribute)
    ```

    ```
     -M
      Treat missing values as an extra value
    ```

    ```
     -P <num>
      Specify the percentage of noise introduced 
      to the data (default 10)
    ```

    ```
     -S <num>
      Specify the random number seed (default 1)
    ```

    :   **Specified by:**: `setOptions` in interface `OptionHandler`
    :   **Parameters:**: `options` - the list of options as an array of strings **Throws:**: `java.lang.Exception` - if an option is not supported

---


### getOptions

```
public java.lang.String[] getOptions()
```

:   Gets the current settings of the filter.

    :   **Specified by:**: `getOptions` in interface `OptionHandler`
    :   **Returns:**: an array of strings suitable for passing to setOptions

---


### useMissingTipText

```
public java.lang.String useMissingTipText()
```

:   Returns the tip text for this property

    :   **Returns:**: tip text for this property suitable for displaying in the explorer/experimenter gui

---


### getUseMissing

```
public boolean getUseMissing()
```

:   Gets the flag if missing values are treated as extra values.

    :   **Returns:**: the flag missing values.

---


### setUseMissing

```
public void setUseMissing(boolean newUseMissing)
```

:   Sets the flag if missing values are treated as extra values.

    :   **Parameters:**: `newUseMissing` - the new flag value.

---


### randomSeedTipText

```
public java.lang.String randomSeedTipText()
```

:   Returns the tip text for this property

    :   **Returns:**: tip text for this property suitable for displaying in the explorer/experimenter gui

---


### getRandomSeed

```
public int getRandomSeed()
```

:   Gets the random number seed.

    :   **Returns:**: the random number seed.

---


### setRandomSeed

```
public void setRandomSeed(int newSeed)
```

:   Sets the random number seed.

    :   **Parameters:**: `newSeed` - the new random number seed.

---


### percentTipText

```
public java.lang.String percentTipText()
```

:   Returns the tip text for this property

    :   **Returns:**: tip text for this property suitable for displaying in the explorer/experimenter gui

---


### getPercent

```
public int getPercent()
```

:   Gets the size of noise data as a percentage of the original set.

    :   **Returns:**: the noise data size

---


### setPercent

```
public void setPercent(int newPercent)
```

:   Sets the size of noise data, as a percentage of the original set.

    :   **Parameters:**: `newPercent` - the subsample set size, between 0 and 100.

---


### attributeIndexTipText

```
public java.lang.String attributeIndexTipText()
```

:   Returns the tip text for this property

    :   **Returns:**: tip text for this property suitable for displaying in the explorer/experimenter gui

---


### getAttributeIndex

```
public java.lang.String getAttributeIndex()
```

:   Get the index of the attribute used.

    :   **Returns:**: the index of the attribute

---


### setAttributeIndex

```
public void setAttributeIndex(java.lang.String attIndex)
```

:   Sets index of the attribute used.

    :   **Parameters:**: `attIndex` - the index of the attribute

---


### getCapabilities

```
public Capabilities getCapabilities()
```

:   Returns the Capabilities of this filter.

    :   **Specified by:**: `getCapabilities` in interface `CapabilitiesHandler` **Overrides:**: `getCapabilities` in class `Filter`
    :   **Returns:**: the capabilities of this object **See Also:**: `Capabilities`

---


### setInputFormat

```
public boolean setInputFormat(Instances instanceInfo)
                       throws java.lang.Exception
```

:   Sets the format of the input instances.

    :   **Overrides:**: `setInputFormat` in class `Filter`
    :   **Parameters:**: `instanceInfo` - an Instances object containing the input instance structure (any instances contained in the object are ignored - only the structure is required). **Returns:**: true if the outputFormat may be collected immediately **Throws:**: `java.lang.Exception` - if the input format can't be set successfully

---


### input

```
public boolean input(Instance instance)
              throws java.lang.Exception
```

:   Input an instance for filtering.

    :   **Overrides:**: `input` in class `Filter`
    :   **Parameters:**: `instance` - the input instance **Returns:**: true if the filtered instance may now be collected with output(). **Throws:**: `java.lang.Exception` - if the input format was not set

---


### batchFinished

```
public boolean batchFinished()
                      throws java.lang.Exception
```

:   Signify that this batch of input to the filter is finished.
    If the filter requires all instances prior to filtering,
    output() may now be called to retrieve the filtered instances.

    :   **Overrides:**: `batchFinished` in class `Filter`
    :   **Returns:**: true if there are instances pending output **Throws:**: `java.lang.Exception` - if no input structure has been defined

---


### addNoise

```
public void addNoise(Instances instances,
                     int seed,
                     int percent,
                     int attIndex,
                     boolean useMissing)
```

:   add noise to the dataset
    a given percentage of the instances are changed in the way, that
    a set of instances are randomly selected using seed. The attribute
    given by its index is changed from its current value to one of the
    other possibly ones, also randomly. This is done with leaving the
    apportion the same.
    if m\_UseMissing is true, missing value is used as a value of its own

    :   **Parameters:**: `instances` - is the dataset: `seed` - used for random function: `percent` - percentage of instances that are changed: `attIndex` - index of the attribute changed: `useMissing` - if true missing values are treated as extra value

---


### getRevision

```
public java.lang.String getRevision()
```

:   Returns the revision string.

    :   **Specified by:**: `getRevision` in interface `RevisionHandler` **Overrides:**: `getRevision` in class `Filter`
    :   **Returns:**: the revision

---


### main

```
public static void main(java.lang.String[] argv)
```

:   Main method for testing this class.

    :   **Parameters:**: `argv` - should contain arguments to the filter: use -h for help


---


|  |  |  |
| --- | --- | --- |
| **Overview** \| **Package** \| **Class** \| **Tree** \| **Deprecated** \| **Index** \| **Help** \| **Weka's home** | |  |
| **PREV CLASS**   **NEXT CLASS** | **FRAMES**    **NO FRAMES**     **All Classes** |  |
| SUMMARY: NESTED \| FIELD \| CONSTR \| METHOD | DETAIL: FIELD \| CONSTR \| METHOD |  |


---
